# Silver-Assisted Hydrogen Evolution from Aluminum Oxidation in Saline Media

**DOI:** 10.3390/molecules29020530

**Published:** 2024-01-21

**Authors:** Olesya A. Buryakovskaya, Konstantin I. Maslakov, Nikita O. Borshchev, Grayr N. Ambaryan, Aleksey V. Butyrin, Mikhail S. Vlaskin

**Affiliations:** 1 Joint Institute for High Temperatures of the Russian Academy of Sciences, 125412 Moscow, Russia; www.moriarty93@mail.ru (N.O.B.); ambaryan1991@gmail.com (G.N.A.); aleksey.butyrin@yandex.ru (A.V.B.); vlaskin@inbox.ru (M.S.V.); 2 Department of Chemistry, Lomonosov Moscow State University, 119991 Moscow, Russia; nonvitas@gmail.com; 3 Algal Research and Bioenergy Lab, Department of Food Science and Technology, Graphic Era (Deemed to be University), Dehradun 248002, India; 4 Department of Environmental Safety and Product Quality Management, Peoples’ Friendship University of Russia, 117198 Moscow, Russia

**Keywords:** aluminum–silver powder, gallium, lithium chloride, high-energy ball milling, aqueous chlorine solution, hydrogen generation

## Abstract

A swarf of aluminum alloy with high corrosion resistance and ductility was successfully converted into fine hydro reactive powders via ball milling with silver powder and either lithium chloride or gallium. The latter substances significantly intensified particle size reduction, while silver formed ‘cathodic’ sites (Ag, Ag_2_Al), promoting Al corrosion in aqueous saline solutions with hydrogen generation. The diffraction patterns, microphotographs, and elemental analysis results demonstrated partial aluminum oxidation in the samples and their contamination with tungsten carbide from milling balls. Those factors were responsible for obtaining lower hydrogen yields than expected. For AlCl_3_ solution at 60 °C, Al–LiCl–Ag, Al–LiCl, Al–Ga–Ag, and Al–Ga composites delivered (84.6 ± 0.2), (86.8 ± 1.4), (80.2 ± 0.5), and (76.7 ± 0.7)% of the expected hydrogen, respectively. Modification with Ag promoted Al oxidation, thus providing higher hydrogen evolution rates. The samples with Ag were tested in a CaCl_2_ solution as well, for which the reaction proceeded much more slowly. At a higher temperature (80 °C) after 3 h of experiment, the corresponding hydrogen yields for Al–LiCl–Ag and Al–Ga–Ag powders were (46.7 ± 2.1) and (31.8 ± 1.9)%. The tested Ag-modified composite powders were considered promising for hydrogen generation and had the potential for further improvement to deliver higher hydrogen yields.

## 1. Introduction

The exploration and wide implementation of renewable, environmentally friendly energy sources remains one of the most crucial modern challenges. The conservation, storage, delivery, and distribution of the harvested geothermal, solar, wind, and marine energy could be provided by the introduction of a secondary energy carrier, such as hydrogen [1,2,3,4,5]. The most remarkable features of that gas are its high heating value (141.8 MJ/kg) and the absence of greenhouse gas emissions from its combustion. Currently, hydrogen is mainly derived from hydrocarbon decomposition, which is expected to be replaced with a cleaner technology—water splitting via electrolysis [6].

The major concerns about the large-scale use of hydrogen are associated with its explosive nature, low density, high diffusivity, and embrittlement of construction materials (e.g., steel and aluminum) induced by this gas [7,8,9,10]. One of the most mature methods for hydrogen conservation is its storage in pressurized form in gas cylinders, whose recent modifications provide quite good storage capacity. Nevertheless, their filling and discharging involve thermal effects that should be accounted for when dealing with flammable and explosive substances. A large amount of heat is released during the fast filling, hydrogen compression in the tank leads to a temperature rise, and, due to the negative Joule–Thomson effect, hydrogen increases in temperature as it expands through the throttle [11,12]. In its liquid form, hydrogen achieves high density, and more energy can be stored per unit volume. However, maintaining an extremely low temperature, considerable energy losses from liquefaction, and gas losses from a boil-off phenomenon make that method too demanding for conventional applications [13,14]. Other rapidly developing hydrogen storage methods are based on hydrogen adsorption and absorption. Carbon nanotubes, metal–organic frameworks, liquid organic hydrogen carriers, complex metal hydrides and intermetallic hydrides, and metal boron–nitrogen–hydrogen compounds keep drawing attention. Their limitations are related to low specific capacity, high sorption/desorption temperatures, and cost and efficiency losses associated with converting the carrier back into hydrogen [15,16].

A simple, scalable solution affording in situ hydrogen generation in a desirable quantity is based on the oxidation of aluminum (or magnesium) in water or aqueous media. Under normal conditions, aluminum has a ‘waterproof’, slowly hydrated, protective oxide layer on its surface [17]. Micron-sized Al powders observably react with distilled water at temperatures from 68–70 °С and higher, while nanoparticles can do that at room temperature [18,19,20]. For that reason, activation measures are needed to promote that process. A well-known approach to boosting the reaction is raising the temperature above 100 °С, which affords effective oxidation of aluminum in both finely dispersed and bulk forms with pure water [21,22,23,24,25,26]. Another ‘timeless’ solution is an alkali solution (such as NaOH, KOH, Ca(OH)_2_, LiOH), which can be effectively applied to aluminum samples with different sizes, compositions, purities, and qualities [27,28,29,30,31,32,33,34,35,36]. Other suitable aggressive corrosive liquids include hydrochloric acid and metal chloride solutions [37,38,39,40,41,42,43]. Other feasible methods are mainly focused on the modification of aluminum composition and structure. That can be done via its melting together with various metals (e.g., Fe, Sn, Cu, Li, Ga, In, Sn) [44,45,46,47,48,49,50,51] or ball milling with salts (e.g., NaCl, KCl, NiCl_2_, CoCl_2_) [52,53,54,55,56], oxides and hydroxides (Bi(OH)_3_, AlOOH, Al(OH)_3_, Al_2_O_3_, TiO_2_, Co_3_O_4_, Cr_2_O_3_, MoO_3_, Bi_2_O_3_, CuO, Cu_2_O) [57,58,59,60,61,62,63], and metals (Cu, Bi, Sn, Pb, Fe, Ni, Zn, Ga, In) [56,64,65,66,67,68,69,70,71]. Experiments with aluminum granules and distilled water under 200–280 °C proved that aluminum vulnerability to oxidation was critically dependent on the amount of impurities in aluminum alloys and their nature [72]. In that study, higher concentrations of Si, Fe, Cu, and Mg in lower-grade alloys (99.7 and 99.9%), as compared to a high-purity alloy (99.99%), and their potential segregation near the surface were considered to be the key factors inhibiting aluminum oxidation. In the Al–Cu–Ga–In–Sn system elaborated in [73], Cu was found to hinder the reaction due to the formation of Al_2_Cu that precipitated at the Al grain boundaries, affecting the distribution of the Ga–In–Sn phase and preventing direct contact between the Al matrix grain and that phase; an increase in the Cu content also led to better refinement of Al grains. In the as-cast and rapidly solidified Al–Mg–Ga–In–Sn alloys manufactured in [74], Mg induced segregation of In and Sn and formation of intermetallic compounds on Al surfaces; for the as-cast alloys, the reaction rates and hydrogen yields lowered with the rise in the Mg content, while in the case of the rapidly solidified ones, the effect was quite the opposite and was associated with Al grain refinement. It is worth mentioning that it is still not clear whether the effect of grain refinement on the corrosion resistance of alloys is positive or negative [75,76,77,78]. According to the results from [79], an increase in the Si content unexpectedly resulted in a decrease in the hydrogen yield; that was attributed to the formation of SiO_2_, which slowed down the elimination rate of the passivation oxide film since it was harder to remove than Al_2_O_3_. In other studies [45,48,80,81,82], however, aluminum alloying with Cu, Fe, Si, and Mg was found to be profitable. From the examples given above, a conclusion could be drawn that the corrosion behavior of aluminum-based materials may vary significantly depending on the concentration, distribution, and specific effects of the alloying additives.

Conventional production of primary aluminum (bauxite mining, Bayer’s process, anode production, electrolysis, and hot rolling) consumes 20.27 kWh of energy and 8.22 kg CO_2_ (equivalent) per 1 kg of metal. Carbon dioxide is primarily emitted from the conventional electricity grid, which can be replaced with a renewable one. Additional decarbonization of the process can be achieved by using inert anodes. Implementation of those measures was estimated to reduce the emissions level to 1.16 kg CO_2_ (equivalent)/kg [83]. Since prices for brand-new aluminum can vary within a wide range, the utilization of waste aluminum with hydrogen generation could represent a viable idea. Aluminum foils, wires, dross, debris, machining products, and dust are considered sustainable materials for hydrogen production [29,84,85,86,87,88,89,90,91,92,93]. Aluminum machining scrap accounts for a significant fraction (13.7%) of the trash created by all manufacturing processes globally [94]. Waste chips and powder of that metal can be effectively ‘converted into hydrogen’ either in their original form or in the form of compacted shapes (e.g., tablets or pellets) [95,96].

Based on the abovementioned considerations, it would be interesting to test an approach to hydrogen generation employing aluminum scrap modification with a nobler metal via ball milling, either gallium or a chlorine salt as a ‘grinding agent’ and carry out the reaction in an aqueous metal chlorine solution. Al modification with Ga results in liquid metal embrittlement of aluminum with its cracking along the grain boundaries. And its ball milling with hard and brittle salts results in a drastic reduction in its particle sizes along with the creation of crystal lattice imperfections, favoring pitting corrosion. And mechanical coupling of Al with nobler metals causes the formation of ‘microgalvanic cells’, enhancing galvanic corrosion of less noble aluminum in conductive media.

In the present study, aluminum swarf chips (also known as shavings) and Ag powder were selected for the preparation of composite hydro reactive powders by high-energy ball milling. In a number of the abovementioned preceding studies, the effects of various metal additives to aluminum (e.g., from commonly used budget-friendly Cu, Fe, and Sn to exotic costly Ga, In, and Li) on its hydro reactive properties were studied. However, no data were found on the implementation of precious metals in order to enhance hydrogen generation from the aluminum–water reaction. In a number of studies [97,98,99], Ag was reported to promote aluminum corrosion in saline media. The corrosion tests in a 3.5% NaCl solution at room temperature revealed pitting areas in the Al matrix around Ag2Al intermetallic precipitates [98,100]. Damaging of the barrier film on the aluminum surface in the vicinity of silver-rich intermetallic sites was also claimed in [101]. And the results from [99] demonstrated the creation of a nanoporous Ag structure via the promoted dissolution of Al from the Al–Ag alloy in a 1 M NaCl solution. Considering the reported results, such an additive is expected to provide an outstanding output. In the experiments, aluminum of A grade (99.99%) will be used in order to eliminate the effects of impurities and focus on the effect of the Ag additive. Tungsten carbide balls, almost twice as dense as stainless-steel ones, will be implemented to provide more intensive mechanical treatment. If the powders obtained by ball milling of the Al–Ag mixture under argon without additives fail to achieve a reasonable particle size reduction or visible oxidation of the components occurs, Ga or anhydrous LiCl will be added as ‘grinding’ agents, and toluene will be used as a process control agent. The effect of solution composition on the reaction rate will be investigated for 2 M aqueous solutions of NaCl, CaCl_2_, and AlCl_3_. The resulting hydrogen yields and evolution rates for different samples will be compared.

## 2. Results and Discussion

### 2.1. Trials for Powder Manufacture

**Figure 1:**
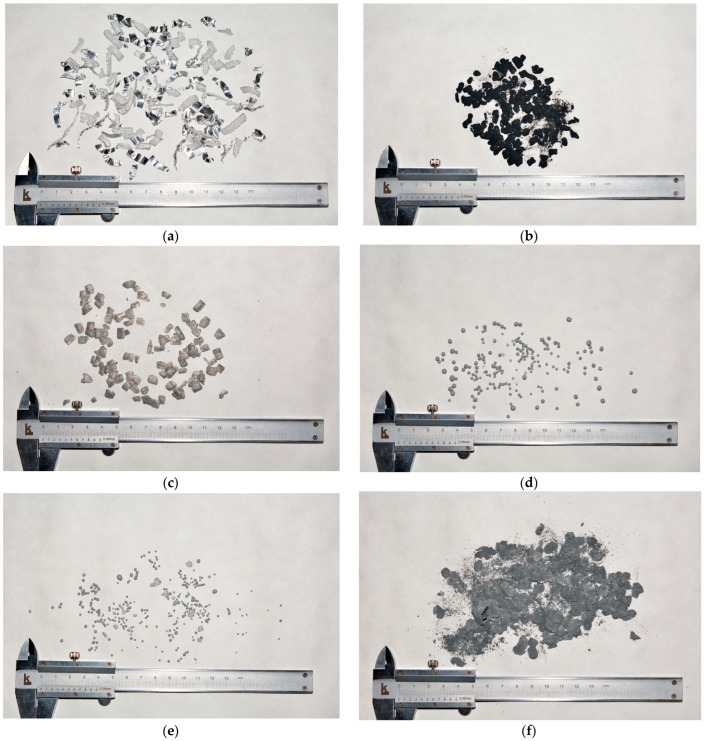

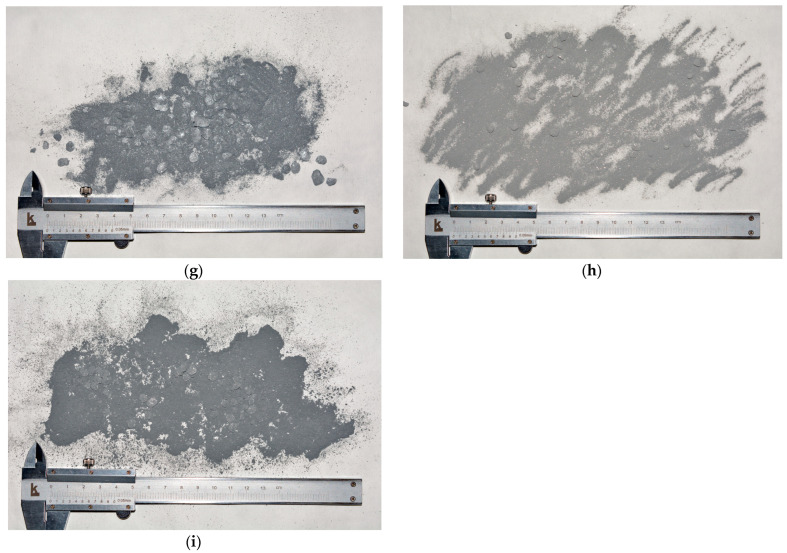
Images of the original and resulting materials: (**a**) aluminum swarf; (**b**) Al–Ag composite (1 h without toluene); (**c**) Al–Ag composite (2 with toluene); (**d**) Al–Ga (1 h without toluene, 15 mm balls); (**e**) Al–Ga (1 h without toluene, 10 mm balls); (**f**) Al–LiCl (1 h without toluene, 15 mm balls); (**g**) Al–LiCl (1 h without toluene, 10 mm balls); (**h**) Al–Ga–Ag (2 h with toluene, 10 mm balls); (**i**) Al–LiCl–Ag (2 h with toluene, 10 mm balls).

In order to manufacture composite Al–Ag powders, several modifications of the ball milling technique were tested. The images of the original aluminum scrap and resulting materials are shown in Figure 1. In a previous study [102], composite powders were successfully produced of D16 alloy (duralumin, similar to the AA2024 grade) and copper powder under the same milling parameters (125 mL milling pot, argon atmosphere, 580 rpm.) with lighter 15 mm balls of stainless steel (balls to powder mass ratio 24:1) after as long as 1 h. In the present study, however, the pioneer sample obtained after 1 h of Al milling together with 10 wt.% Ag under Ar with a set of 15 mm tungsten carbide balls (almost twice as heavy as those of steel) represented large chips cold-welded to each other and covered with black powder. Apparently, despite intense purging with argon, the amount of residual air in the milling pot appeared to be sufficient to oxidize silver at an elevated temperature caused by the heat released from the high-speed collisions between heavy balls. Taking that into account, the next sample was prepared via longer (2 h) milling with the addition of 1.25 mL of toluene as a process control agent to prevent both cold welding of aluminum pieces and oxidation of silver particles. Although no visual evidence of silver oxidation and cold welding was observed, the swarf particles were flattened but still too large in size, and the silver particles did not look attached to them. Heavier balls were expected to accelerate the structural evolution of aluminum particles: flattening, accumulation of microstrains and embrittlement, and further progress of two competing processes of fracturing in the hardened regions and agglomeration of the resulting smaller pieces caused by cold welding (first, into flattened lamellar structures, and finally, into compacted equiaxed solid shapes) [61,103,104,105]. However, in the present study, the base material was represented by quite large pieces, several mm in size. Furthermore, even when strain hardened, highly refined aluminum was characterized by extremely high ductility and formability along with low strength, in contrast with the Al–Zn and Al–Cu series of alloys, e.g., ‘AA 7075’ and ‘AA 2024’ grades, characterized with high hardness (due to precipitation hardening) and significant size reduction during ball milling [106,107,108,109].

For the mentioned reasons, the next step towards powder elaboration was testing additives that could promote particle size reduction. For that purpose, two alternatives were tried. The first step was the addition of 20 wt.% anhydrous LiCl to aluminum powder to promote ‘cutting’ of its ductile particles with hard, brittle salt pieces. That salt was selected because it was anhydrous and, therefore, was not expected to provide aluminum oxidation with bonded water during ball milling. Also, it was insoluble in toluene but soluble in ethanol and acetonitrile (with the potential to be removed by dissolution in those liquids without aluminum oxidation). Another alternative was the addition of 1 wt.% Ga to provide aluminum embrittlement—a manifestation of the Rehbinder effect caused by the impact of mechanical stress and adsorption-active liquid metal. The anticipated result was enhanced aluminum fragmentation during ball milling, avoiding the potential formation of Ag_2_Ga at elevated temperatures [110]. Trials of either of those additives included their milling with Al scrap for 1 h under Ar atmosphere using a set of ‘big’ (15 mm) or ‘small’ (10 mm) tungsten carbide balls under the same ball-to-powder mass ratio of 47:1. In the case of LiCl, disperse materials were obtained; however, the ‘big’ balls provided worse particle size reduction as a lot of large flattened flakes were not ground into powder. The addition of Ga resulted in the formation of equiaxed solid objects with almost spherical shapes (again, larger for ‘big’ balls) that could be attributed to a drastic acceleration of ‘particle structural evolution’ during ball milling from the combination of intense fracturing and cold-welding processes.

Summarizing the abovementioned results, the powders for the experiments were prepared by adding 1.25 mL of toluene, sealing the milling pot under Ar, and 2 h of ball milling with ‘small’ (10 mm) tungsten carbide balls. For the experiments, the following powder compositions were prepared: Al–20 wt.% LiCl, Al–20 wt.% LiCl–10 wt.% Ag, Al–1 wt.% Ga, and Al–1 wt.% Ga–10 wt.% Ag (units in wt.% are fractions of Al mass in the samples). In the respective images, only samples with Ag were depicted (samples without Ag had a similar look). Eventually, rather fine powers were obtained.

### 2.2. Phase Composition

**Figure 2:**
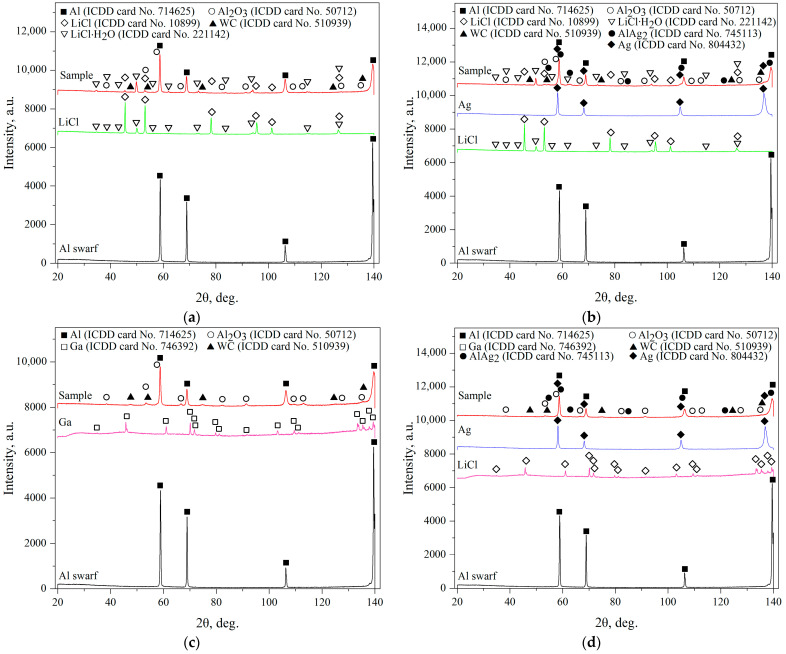
XRD patterns for different samples: (**a**) Al–LiCl; (**b**) Al–LiCl–Ag; (**c**) Al–Ga; (**d**) Al–Ga–Ag.

The XRD patterns for the ball-milled composite powders are shown in Figure 2. The high peaks for the Al swarf sample were attributed to its texturization, which apparently resulted from machine processing. Although the LiCl sample was claimed to be anhydrous and was subjected to drying prior to the analysis, its patterns revealed the presence of a hydrated phase, LiCl∙H_2_O. The same phase was observed in the patterns for the respective powder samples. Probably, hydration took place during the preparation of the samples for scanning. No gallium peaks were identified in the samples because of their low content (obviously, 1 wt.% fell beyond the detection limits of the diffractometer). The samples modified with Ag demonstrated the existence of Ag and AlAg_2_ phases (the formation of the latter was apparently caused by intensive impacts and heat release). It turned out that all of the samples were contaminated with WC from the milling balls (which were probably designed for slower rotation). It was evident that, despite all precautions (intense Ar purging and addition of toluene), air somehow reached aluminum, which, under elevated temperature, was oxidized with the formation of Al_2_O_3_, and the samples with Ga generally had larger Al_2_O_3_ peak areas than those with LiCl. Probably, further improvements (tighter sealing, implementation of a lid with valves for direct Ar purging of the milling pot instead of the glove box, etc.) should be included in the preparatory procedures.

### 2.3. Microstructure, Specific Surface Area, and Elemental Composition

**Figure 3:**
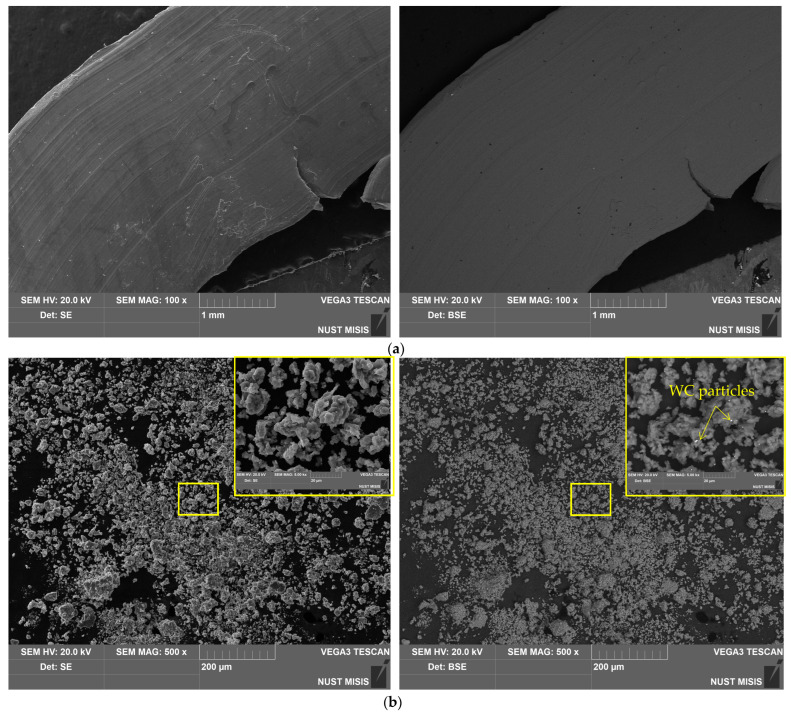

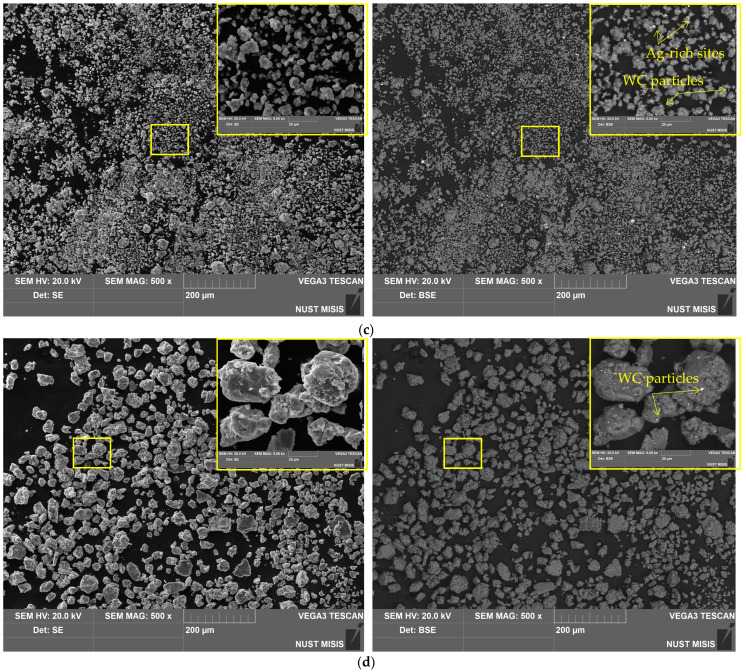

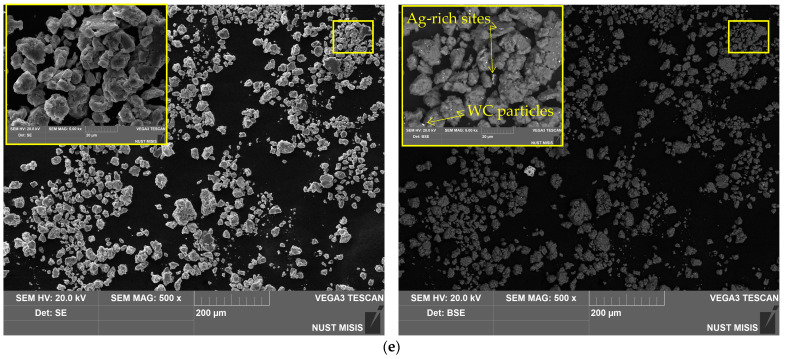
SEM images (SE **leftward** and BSE **rightward**) for different samples: (**a**) aluminum swarf; (**b**) Al–LiCl; (**c**) Al–LiCl–Ag; (**d**) Al–Ga; (**e**) Al–Ga–Ag.

The microstructures of the original aluminum swarf and composite powders are illustrated in Figure 3. The depicted piece of aluminum swarf had traces of machining (grooves, rough edges, bends, and cuts). The powders containing LiCl had notably finer particles than those with Ga. Therefore, under the tested milling conditions, better refinement was achieved by intensive mechanical grinding with 20 wt.% of the salt rather than via fracturing induced by the Rehbinder effect in the presence of 1 wt.% Ga. The particles mostly represented agglomerates of smaller pieces that formed from the combination of the abovementioned processes of fracturing (in the case of Ga) or disintegration by cutting (for LiCl) with cold welding under high-energy collisions between the milling balls.

The specific surface area was measured for the Al–LiCl–Ag and Al–Ga–Ag samples by the low-temperature nitrogen sorption method. According to the results, the first powder achieved 2.105 m^2^/g, which was considerably higher than 0.146 m^2^/g for the second one. Those data were generally in agreement with the results of the powders’ inspection via SEM: Smaller particles containing LiCl provided a more extended surface.

The base material of the particles was depicted in gray shades (from dark to light ones), while the inclusions of heavy metals (Ag, W) were depicted in whitish and white colors. The tiny white spots with sharp, edgy contours more likely corresponded to WC chipped-off from the milling balls in the form of small particles, which, due to their hardness, were ‘incrusted’ into the ductile metal without significant deformations of their shape. The larger whitish areas with blurry outlines represented the sites enriched with Ag that, due to its high ductility, could be ‘merged’ with aluminum.

**Figure A1:**
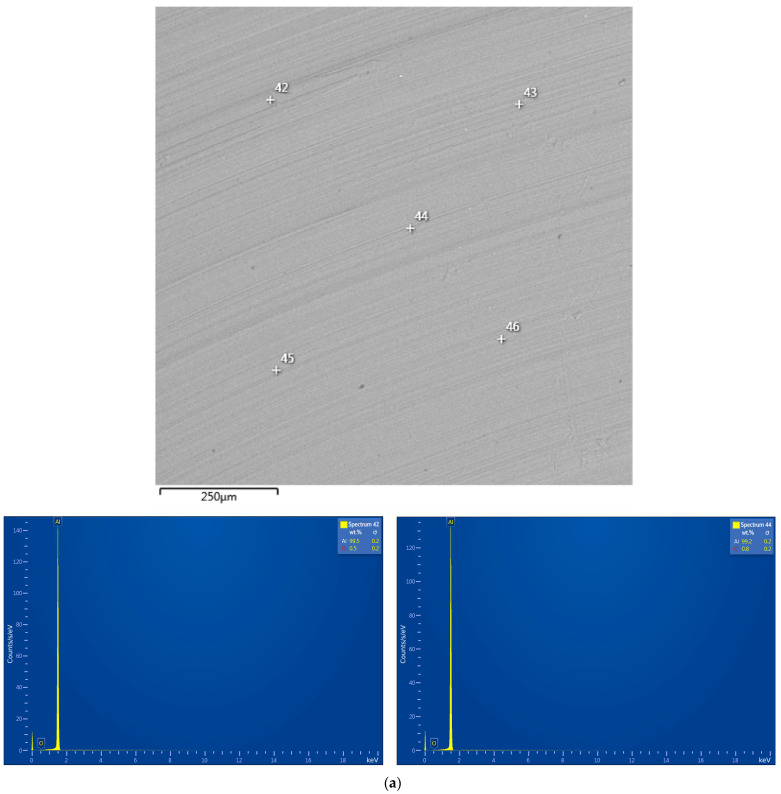

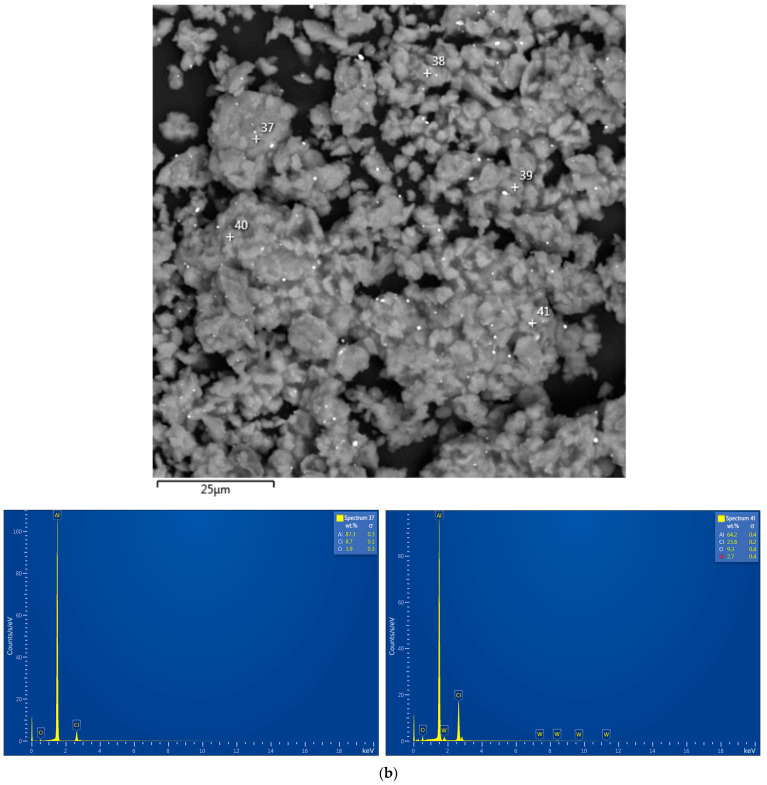

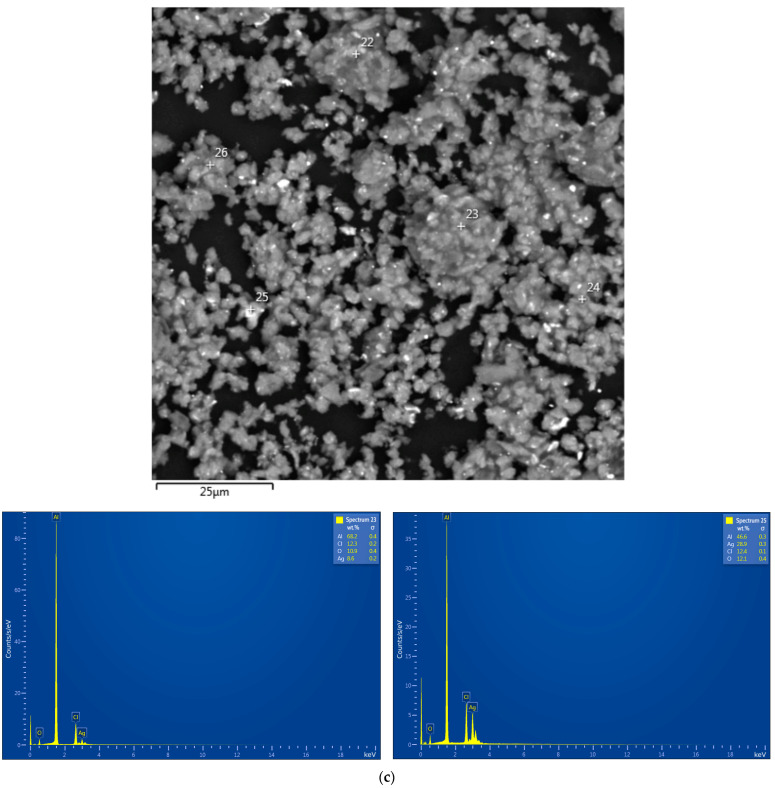

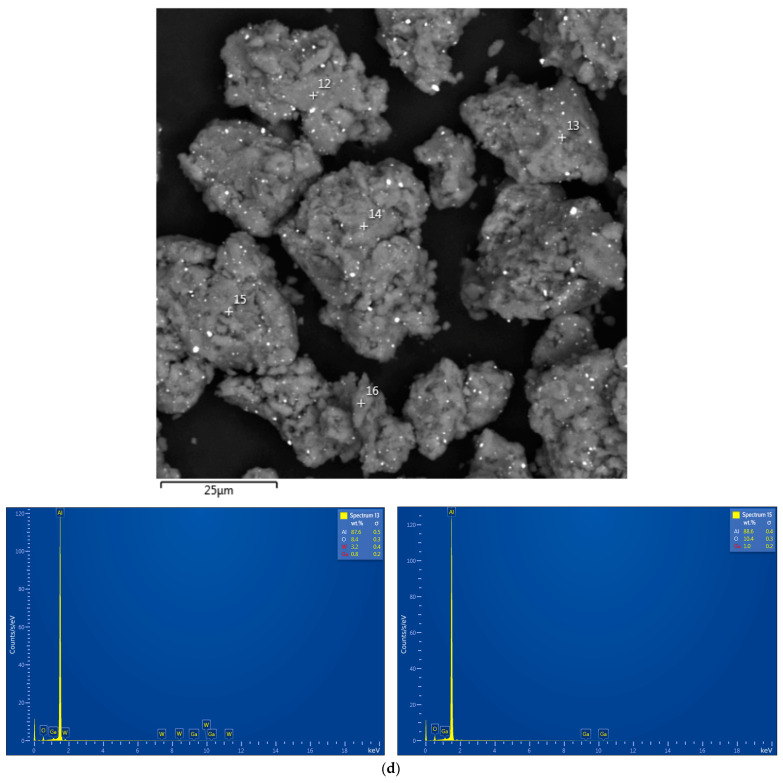

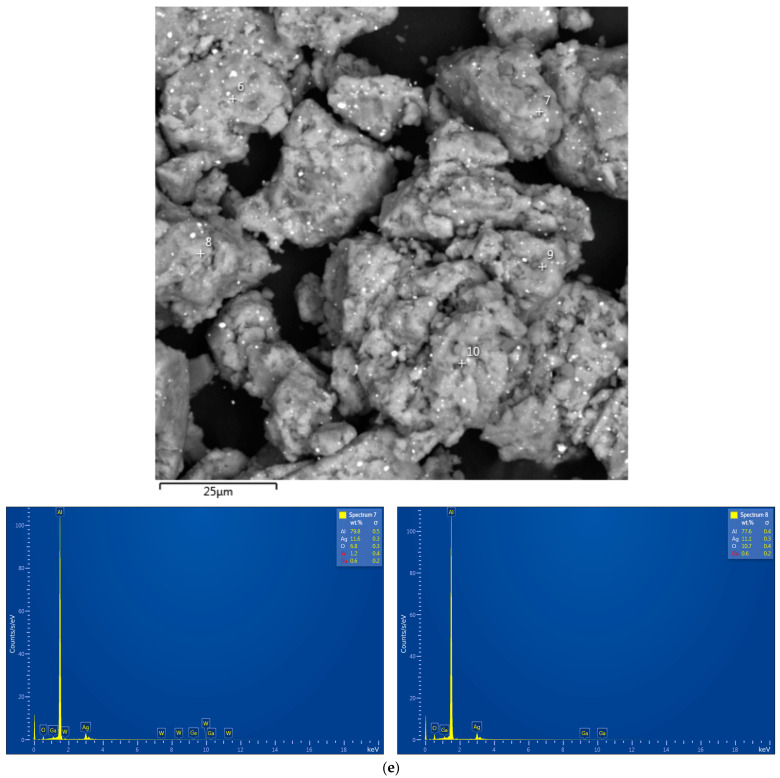
EDX analysis results (spectra) at the selected points for the starting material and composite powders: (**a**) aluminum alloy swarf; (**b**) Al–Li; (**c**) Al–Li–Ag; (**d**) Al–Ga; (**e**) Al–Ga–Ag.

The output from the EDX analysis is illustrated in Figure A1 (see Appendix A) and summarized in Table 1, wherein the elemental compositions for the selected scanned points are listed. For the aluminum swarf, no elements other than aluminum and a minor (up to 0.8 wt.%) amount of oxygen were detected. Thus, no significant oxidation of the starting material took place, and the original aluminum was almost ‘oxygen-free’. The powder samples obtained by milling with LiCl demonstrated the presence of Cl. Most of the currently used EDX setups were not intended for the detection of Li: Its low X-ray signal and high probability of absorption by the detector window resulted in a small emitted intensity [111]. In the present study, Li fell beyond the detection limits as well. In contrast to the XRD analysis results, the EDX data proved the presence of Ga in the respective samples. For the powder samples, their enrichment with Ag and contamination with WC were demonstrated. Also, the EDX analysis output confirmed that the powder samples contained relatively high contents of oxygen, which was in agreement with the XRD analysis results. Obviously, during high-energy ball milling with heavy tungsten carbide balls, the conditions favoring Al oxidation with residual air were established, and the ‘grinding’ effects of Li and Ga increased the intensity of that process. However, oxygen quantification by the EDX method is not reliable since it has low-energy X-ray emission that can be absorbed by the specimen or the detector window [112]. Moreover, the implementation of carbon surface coatings makes quantification of carbon infeasible [113], while the XRD analysis results proved its presence in the samples. For those reasons, no precise data on the light element content in the samples were obtained from the EDX investigation.

**Figure 4:**
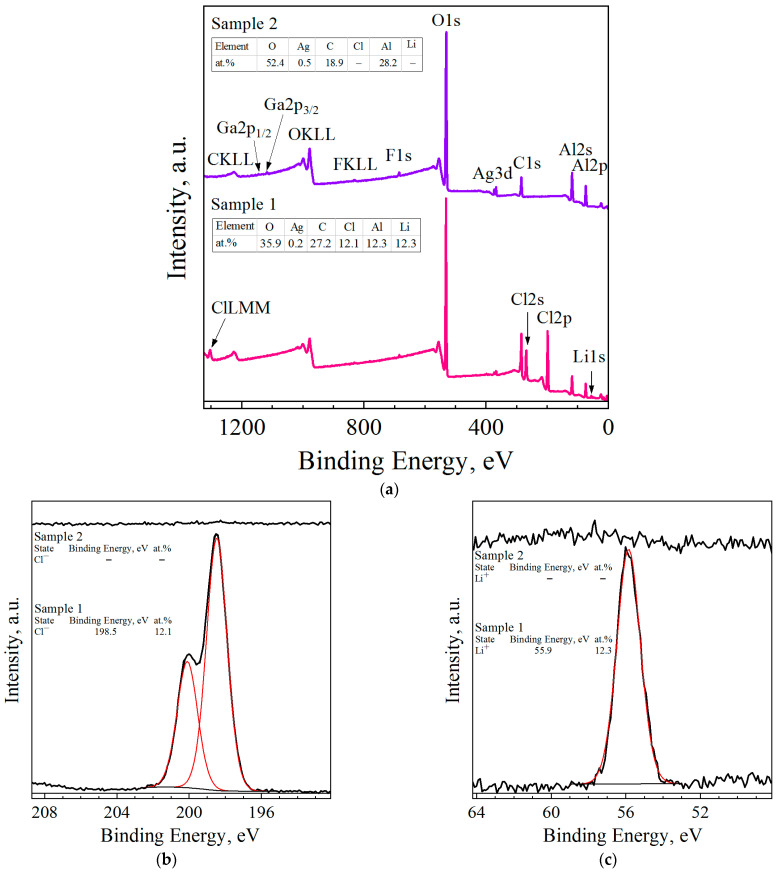

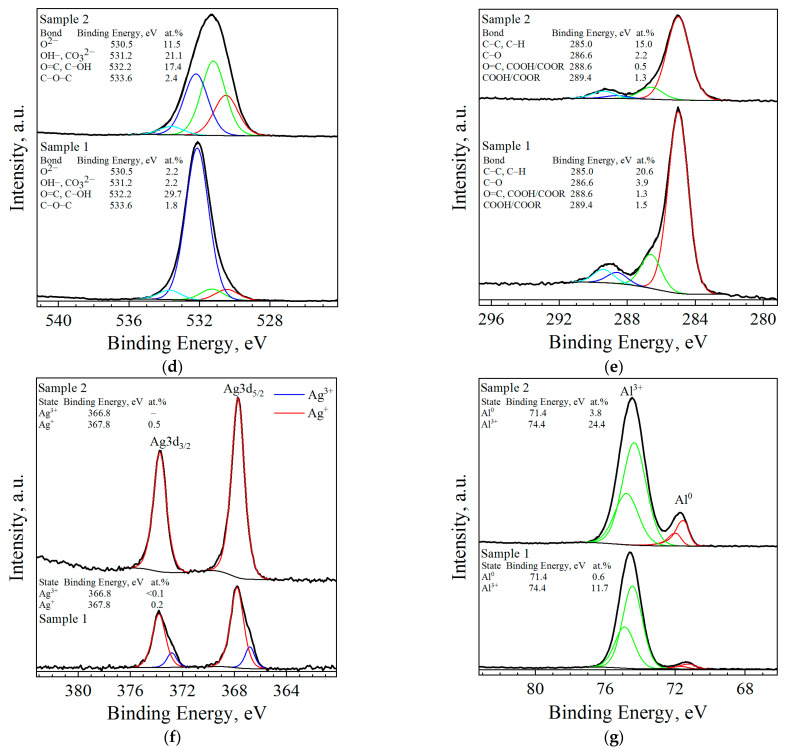
XPS analysis data for ball-milled samples (sample 1—Al–LiCl–Ag; sample 2—Al–Ga–Ag): (**a**) XPS survey scans of the surfaces of the samples; (**b**) Cl2p peak fitting; (**c**) Li1s peak fitting; (**d**) O1s peak fitting; (**e**) C1s peak fitting; (**f**) Ag3d peak fitting; (**g**) Al2p peak fitting.

The XPS survey scanning of the surfaces of the Al–LiCl–Ag (sample 1) and Al–Ga–Ag (sample 2) composites revealed the characteristic peaks of Al, O, C, Ag, and faint lines of F (see Figure 4). For the first sample, considerable amounts of Li and Cl were detected as well, while for the second one, faint lines of Ga were observed. Binding energy calibration was carried out by fixing the adventitious C1s peak (285.0 eV, maximum for C–C/C–H bonds) as an energy reference; that peak corresponded to carbon contamination adsorbed on the samples’ surface. The resulting high-resolution spectra peaks (represented in Figure 4b–g) were deconvoluted into a number of patterns corresponding to different atomic states of the identified elements. The XPS spectrum for Al2p revealed the predominance of the oxidized state (Al^3+^) over the metallic aluminum (Al^0^). In the Ag3d spectrum of the Al–LiCl–Ag sample, for the Ag3d_5/2_ component, two states with binding energies of 366.8 and 367.8 eV were identified, which was less than the binding energy of that line in pure metallic silver (368.2 eV [114]). The observed binding energies were recognized as those for the Ag^3+^ and Ag^+^ states, respectively [115], and those detected for the Li and Cl lines were typical for the respective ions presenting in different salts [116]. The spectra of the O lines (especially for the Al–Ga–Ag sample) were wide, which could be attributed to the presence of various states of oxygen at the surface. Those states probably resulted from the formation of oxides, hydroxides, and carbonates onto the aluminum surface, as well as carbon-containing contaminants.

The tables with the concentrations of the elements detected on the samples’ surfaces, inserted in the XPS survey scan, are shown in Figure 4a. The XPS analysis results proved the presence of the light elements (C, O, and Li), which were not detected by EDX analysis. The numerical data proved the high content of oxygen on the samples’ surfaces. Its content was higher in the sample with LiCl than in that with Ga. Some carbon contamination was observable as well.

### 2.4. Reaction Kinetics

**Figure 5:**
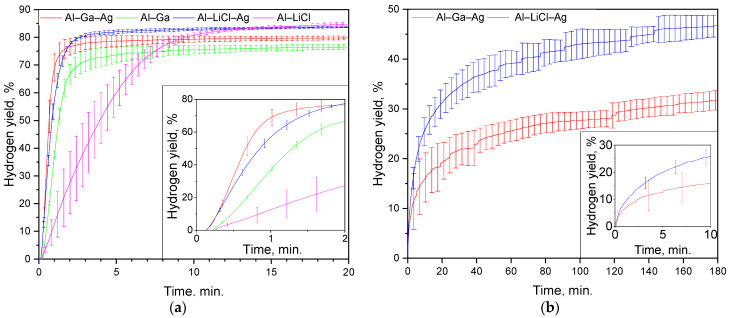
Hydrogen evolution kinetic curves: (**a**) powder samples in 2 M AlCl_3_ solution at 60 °C; (**b**) Al–Ga–Ag and Al–LiCl–Ag samples in 2 M CaCl_2_ solution at 80 °C.

The experimental results on the hydrogen evolution kinetics for the composite powder samples are depicted in Figure 5. As it can be seen from the plot for the AlCl_3_ aqueous solution (only the first 20 min. of the experiments lasting for 1 h are depicted), all kinetic curves had a short acceleration section at the beginning, followed by a steep uprise that was gradually changed for a deceleration portion, and a plateau. The highest hydrogen evolution rate of 792 mL/g/s corresponded to the Al–Ga–Ag sample, while the second- and third-fastest reactions were observed for Al–LiCl–Ag and Al–Ga samples (586 and 588 mL/g/s, respectively), and the slowest process (183 mL/g/s) took place in the case of the Al–LiCl composite (see Table 2). It was revealed that the samples modified with Ag demonstrated considerably higher reaction rates as compared to the samples with the same ‘grinding agent’ (LiCl or Ga), as expected. Another notable observation was that the samples with LiCl achieved a higher degree of aluminum ‘conversion into hydrogen’ as compared to those milled with Ga. The final hydrogen yields after 1 h of experiment achieved (80.2 ± 0.5), (76.7 ± 0.7), (84.6 ± 0.2), and (86.8 ± 1.4)% for Al–Ga–Ag, Al–Ga, Al–LiCl–Ag, and Al–LiCl samples, respectively. The obtained results could be explained based on the sample analysis results.

Presumably, for the powders with Ga, more severe oxidation of aluminum took place during ball milling, leading to its lower content in the samples, while in the case of LiCl, after some time, aluminum particles could, in contrast, get partially protected with the salt particles attached to their surfaces. Such an assumption was supported by the results of XRD analysis (slightly larger Al_2_O_3_ peak areas for the samples containing Ga as compared to those with LiCl pointed to higher aluminum oxide contents) and XPS data (considerable amounts of Li and Cl on the surface of the salt-containing sample). It should be noted that the above assumption was derived from the contribution of all oxidized aluminum (not only oxidized surface layers). Also, it was notable that, for the aluminum samples with the same ‘milling agent’, their ‘preoxidation degrees’ did not significantly and similarly differ from each other by the presence or absence of Ag. Therefore, the ‘preoxidation’ was seemingly caused by the impact of the ‘milling agents’, while Al coupling with Ag barely boosted that process. The contamination of the samples with WC from the milling balls also reduced their ‘consumable’ aluminum contents since weighing the samples prior to experiments was carried out without taking that factor into account.

The version explaining the deviations in the reaction rates could be the following. Although the powders with LiCl obviously had significantly finer particles (that was proved by both the specific surface measurements and microphotographs) and, therefore, were anticipated to have a larger specific surface area, the presence of the said incrusted salt particles could potentially hinder aluminum oxidation at the beginning as their dissolution might take some time. Such an idea was in agreement with the XPS data, which demonstrated considerable amounts of Li and Cl on the surface of the Al–LiCl–Ag sample. Also, that analysis revealed that the Al–LiCl–Ag sample also had a higher O content on its surface, which could delay the reaction beginning as well. And the positive impact of Ag on the reaction rates of the corresponding samples lies in the fact that the mechanical coupling of Al with the nobler silver resulted in the formation of ‘microgalvanic cells’ that enhanced galvanic corrosion of the less noble aluminum in conductive media (salt aqueous media) that was accompanied by vigorous hydrogen evolution.

Another set of experiments was carried out with the most ‘promising’ samples, modified with Ag, which were tested at 60 °C in 2 M NaCl and CaCl_2_ solutions. The respective results fell far beyond expectations since no observable reaction progress was detected under those conditions. Raising the temperature up to 80 °C made a difference; however, for the NaCl solution, the kinetics was still quite unimpressive (nearly 11% hydrogen was released in 4 h). Considerably better results were obtained for the CaCl_2_ media (see Table 3). The reaction started immediately, in contrast with the case of AlCl_3_, and no acceleration sections were recognized at the beginning (however, that probably could result from the peculiarities of the measurement system that needed to reach a slight overpressure to start ejecting water or to restart that operation after a pause). The maximum hydrogen release rates were generally comparable (that was confirmed with the overlapping error bars of the respective kinetic curves), 72 and 89 mL/g/s for the powders with Ga and LiCl, respectively. However, before achieving high outputs, the trends turned towards deceleration. Three hours of experiments yielded as much as (46.7 ± 2.1) and (31.8 ± 1.9)% hydrogen for the Ag-modified samples with LiCl and Ga, respectively. The reaction actually continued its progress at the moment of the experiment termination, albeit it progressed very slowly. Along their deceleration portions, the kinetic curves had distinguishable stairstep shapes. In studies [34,35,36], the process of Al oxidation in Ca(OH)_2_ solutions was reported to start with rapid consumption of OH^−^ ions with vigorous hydrogen release, then pause due to passivation, and then, after a while, restart due to reactivation. In the present study, such a peculiarity could arise from the consideration that the CaCl_2_ solution did not support the continuous formation of soluble Al-based compounds, and the formation of a dense reaction product layer onto the particles’ surfaces took place. After that, it took some time for the ‘encapsulated’ aluminum to be reached by liquid media via its diffusion through the poorly permeable reaction product layer and, probably, small pitting corrosion sites and for the emerging hydrogen bubbles to gain pressure to break through the shells. In more detail, the difference between the effects of the tested solutions is discussed below.

The pH values of the 2 M AlCl_3_, CaCl_2_, and NaCl solutions measured at 25 °C with a Multiparameter Transmitter M300 (JSC ‘Mettler Toledo’, Greifensee, Switzerland) were 1.25 ± 0.03, 6.21 ± 0.03, and 6.87 ± 0.03. The beneficial effect of AlCl_3_ consists in its hydrolysis into highly soluble complexes: Al(H_2_O)_6_^3+^ (pH < 3.6), [Al(OH)(H_2_O)_4_]^2+^ and [Al(OH)_2_(H2O)_2_]^+^ (pH~4), [Al(OH)(H_2_O)_4_]^2+^ and [Al(OH)_4_]^−^ (5.2 < pH < 6.7), and [Al(OH)_4_]^−^ (pH > 7.0) [117,118]. According to those data, the measured pH corresponded to the Al(H_2_O)_6_^3+^ soluble compound, i.e., the hydrolysis of the AlCl_3_ salt delivered rather a large amount of chlorine ions, which are known to promote aluminum corrosion [119,120]. In studies [121,122], CaCl_2_ was reported to be hydrolyzed with the formation of [CaOH]^+^; the fraction of the ‘released’ chlorine ions, however, was far less than that for AlCl_3_. And the NaCl solution was subjected to negligible hydrolysis at most. As discussed in a previous study [102], the reaction between aluminum samples and aluminum chloride solution with hydrogen evolution resulted in the formation of the hydroxychloride compound Al_m_(OH)_n_Cl_3m−n_ (m ≥ 1; 0 < n ≤ 3m). Compared to the typical reaction products, AlOOH and Al(OH)_3_, such complex compounds are characterized by a much higher solubility in water. The replacement of a dense, poorly permeable layer of the reaction product depositions isolating the particle surfaces from aqueous media with the reaction product resulted in its continuous removal by dissolving in the solution itself. And that was a crucial factor that ensured continuous aluminum oxidation with hydrogen generation. The impact of the other tested solutions, CaCl_2_ and NaCl, presumably consisted in the clustering of the less numerous chloride ions near the structural imperfections in the Al crystal lattice (e.g., vacancies, voids, dislocations, grain boundaries, inclusions, and second-phase particles [123]) that caused localized destruction of the poorly soluble passivation layer of the conventional reaction products via pitting corrosion mechanisms.

The inspection of the long-term stability of the samples provides valuable information about their exploitation properties. Continuous exposition to air and even storage in argon or nitrogen environments were reported to result in the degradation of their hydrogen generation properties (aging) due to the formation of aluminum oxide over the surfaces exposed to the air environment and a decrease in the microstructure imperfections due to recovery, recrystallization, or grain growth processes [124,125,126]. In the present study, however, despite extensive purging with argon and the implementation of toluene, the ‘fresh’ samples already contained an observable amount of oxygen (that was proved by X-ray diffraction analysis, energy-dispersive X-ray spectroscopy, and X-ray photoelectron spectroscopy). Such an issue was not unique: in a number of preceding studies, high-energy ball milling was reported to cause partial oxidation of the hydro reactive material (magnesium or aluminum) with residual or leaking air or with residual oxides present at the surface of the milling tools [127,128,129]. Preventing aluminum from oxidation during milling should be ensured. To eliminate that ‘preoxidation effect’, additional efforts on the milling equipment and process optimization should be applied. For instance, a lid with nozzles could be manufactured for the milling pot to ensure its better purging with argon. Probably, a larger amount of toluene might be helpful. Lower rotational speed in combination with longer ball milling could be tested as well in order to reduce energy release per impact while ensuring appropriate particle size reduction. Upon ensuring ‘oxygen-free’ ball milling mode (or one close to that), the samples should be tested for long-term stability under air and argon atmospheres.

From the summarization of the key ideas stated above, the following major conclusions can be drawn. All samples (with Ag and without it) lacked roughly 10–20% of the expected hydrogen yield. Since the high-energy ball milling was performed using a set of heavy tungsten carbide balls, the collisions between them were quite powerful and resulted in a large heat release per impact. That, together with the ‘size-reducing effects’ (i.e., exposing extra aluminum surface) of lithium chloride or gallium, led to an observable oxidation of the original aluminum during milling. The powders modified with Ag demonstrated high hydrogen evolution rates. The achieved hydrogen yields were lower than expected due to the abovementioned oxidation effect and, to a minor extent, due to the contamination with tungsten carbide pieces since a smaller fraction of unoxidized aluminum, capable of reacting with hydrogen generation, remained in the samples. In the experiments with the AlCl_3_ solution, fast reaction progress was attributed to its almost complete hydrolysis, which delivered a lot of chloride ions. The chloride ions provided the formation of complex compounds with high solubility in the solution, which ensured their continuous removal from the samples’ surfaces. In the case of the CaCl_2_ ‘brine’, an incomparably smaller amount of chlorine ions was formed from its hydrolysis, and their potential impact consisted in clustering in the vicinity of the aluminum crystal lattice imperfections and local destruction of the passivation layer by the pitting corrosion mechanism.

Although the hydrogen production performance of the tested composite materials turned out to fall beyond initial expectations, they seemed to have potential for improvement. It should be noted that the corrosion resistance of 1xxx aluminum alloy grades is so strong that those materials can be applied in direct contact with seawater and antiskid salts [109]. Therefore, some other scraps of aluminum could be more vulnerable to oxidation with the same saline solutions with hydrogen evolution and have lower ductility, which is beneficial for particle size reduction during milling. The procedure for the ball milling of aluminum with Ga and Ag or LiCl and Ag should be upgraded so as to avoid aluminum oxidation. For the powder particles, additional investigation of their cross sections by SEM-EDX techniques could be recommended to establish their internal structure and elemental distribution, as it was performed in studies [55,61,124]. To recover the precious metal (silver) from the reaction product/solid residuals, density separation techniques with heavy liquids could be tested [130].

## 3. Materials and Methods

The key components of the composite powders were chemically pure aluminum (99.99 wt.%, Technical Specification No. 4-271-10, ‘Component-Reaktiv’ Ltd., Moscow, Russia) in the form of swarf chips, whose composition was similar to that of the ‘AA 1199’ aluminum alloy grade, and silver powder ‘PS-3’ (99.85 wt.%, Technical Specification No. 1752-001-59839838-2003, ‘NPP Delta-Pasty’ Ltd., Moscow, Russia). Extra pure grade gallium (99.999 wt.%, ‘Osobo Chistye Veschestva’ shop, sole proprietorship, Moscow, Russia) and pure anhydrous LiCl (>99.0 wt.%, Technical Specification No. 2-476-11, ‘Component-Reaktiv’ Ltd., Moscow, Russia) were employed as assisting agents in the ball milling, and grade A petroleum toluene (>99.75 wt.%, National Standard GOST 14710-78, ‘ReaKhimLab’ Ltd., Moscow, Russia) was used as a process controlling agent. Aqueous salt solutions with a concentration of 2 M were prepared of distilled water and the following reagents: chemically pure NaCl (National Standard GOST 4233-77, ‘LabTech’ Ltd., Moscow, Russia), pure anhydrous CaCl_2_ (Technical Specification No. 2-239-10, ‘Component-Reaktiv’ Ltd., Moscow, Russia), and chemically pure AlCl_3_∙6H_2_O (Technical Specification No. 2-191-10, ‘Component-Reaktiv’ Ltd., Moscow, Russia).

The samples of composite materials were manufactured using a centrifugal ball mill (S 100; ‘Retsch’ GmbH, Haan, Germany) and a 125 mL milling pot filled in a glove box (G-BOX-F-290; ‘FUMATECH’ Ltd., Novosibirsk, Russia) under pure argon (99.993%, National State Standard GOST 10157-79, ‘NII KM’ Ltd., Moscow, Russia). Heavy tungsten carbide balls 15 and 10 mm in diameter were tested under a ball-to-powder ratio of 47:1 and a rotation speed of 580 rpm. Large particles (exceeding 1000 μm) were separated from the fine powders via a sieve shaker (SS 207/B09, ‘Technotest’ S.r.l., Modena, Italy). The volatile agent (toluene) was removed from the powders by drying with argon in the glove box.

Prior to the analyses, some of the tested materials (all composite powders and LiCl salt) were dried at 70 °C in a drying oven (SNOL 24/200, JSC ‘Umega’, Utena, Lithuania). The X-ray diffraction (XRD) analysis of the original materials (aluminum swarf, silver powder, gallium, and lithium chloride) and ball-milled composite powders was performed at a ‘Difraey 401’ diffractometer (‘Scientific Instruments’ JSC, Saint Petersburg, Russia) with Cr-Kα radiation (0.22909 nm). The scanning was performed under the Bragg–Brentano focusing geometry in the 2θ angular range from 20 to 140° (step size 0.01°). The XRD patterns were identified using the PDF-2 database (Powder Diffraction File™) from the International Centre for Diffraction Data (ICDD). The microstructure of the samples was investigated by the scanning electron microscopy (SEM) method in the secondary electron (SE) and backscattered electron (BSE) modes. The elemental compositions of the samples were analyzed by the energy-dispersive X-ray spectroscopy (EDX) method under a 20.0 keV operating voltage. The SEM-EDX investigation was conducted via a scanning electron microscope (TESCAN VEGA3, ‘Oxford Instruments’ PLC, Abingdon, UK). And the images depicting a general view of the original material and samples were taken by a digital camera (Nikon D5200, ‘Nikon Corporation’, Shanghai, China) with the lens (AF-S DX Micro Nikkor 40 mm f/2.8G, ‘Nikon Corporation’, Shanghai, China). The specific surface area measurements were conducted using a surface area and pore size analyzer Nova 1200e (‘Quantachrome Instruments’ LLC, Boynton Beach, FL, USA). The results from low-temperature nitrogen sorption measurements were processed via ‘Quantachrome NovaWin’ software (version 10.0), applying the Brunauer–Emmett–Teller (BET) equation. And the top-surface chemistry analysis was performed via the X-ray photoelectron spectroscopy (XPS) method using an X-ray photoelectron spectroscope (Axis Ultra DLD, ‘Kratos Analytical’ Ltd., Manchester, UK), with monochromatic AlKα radiation. The pass energy of the analyzer was 160 eV for survey spectra and 40 eV for high-resolution scans. ‘Casa XPS’ software (version 2.3.23) was used for spectra processing. The precision of the binding energy calculation was 0.1 eV.

**Figure 6:**
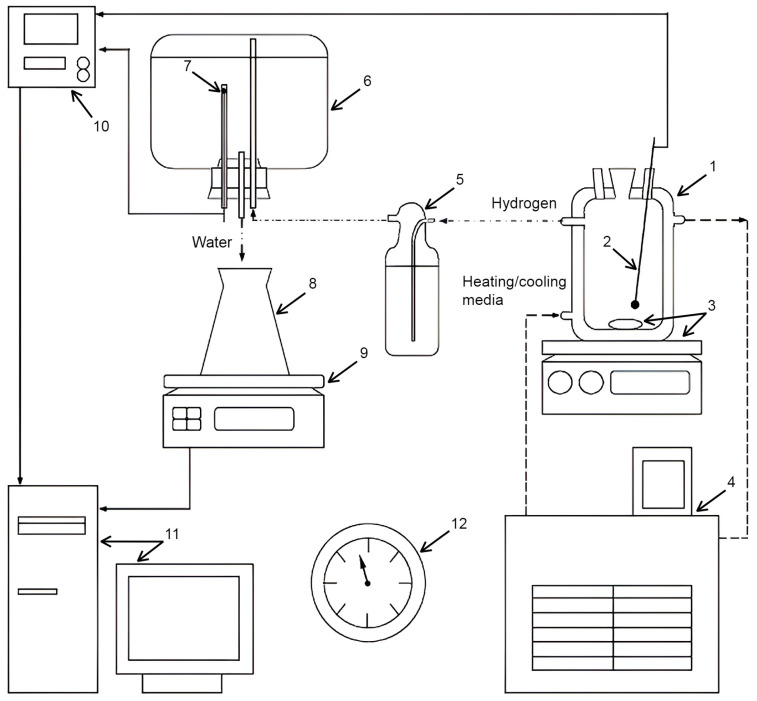
Compacted pellets and experimental set: 1—reactor; 2—thermocouple; 3—magnetic mixer and stirring bar; 4—thermostat; 5—Drexel flask; 6—glass vessel; 7—resistance temperature detector; 8—flask; 9—scales; 10—multichannel thermometer; 11—computer; 12—barometer [43].

The experiments were carried out in a reactor (1000 mL, JSC ‘Lenz Laborglas’, Wertheim, Germany) filled with 1000 mL of the solution under stirring (300 rpm) with a magnetic mixer (C-MAG HS 7; JSC ‘IKA-Werke’, Staufen, Germany). The isothermal regime was maintained via a heater (CC-308B; JSC ‘ONE Peter Huber Kältemaschinenbau’, Offenburg, Germany). The mass of the powder samples was (0.5000 ± 0.0003) g. Hydrogen evolving during the experiments was delivered through a Drexel flask into a glass vessel with water, which was ejected into a flask positioned onto scales (ATL-8200d1-I; ‘Acculab Sartorius Group’, New York, NY, USA). The mass readings were transmitted to a computer in continuous mode. The temperatures in the reactor and glass vessel were measured correspondingly with an L-type thermocouple (TP.KhK(L)-K11; ‘Relsib’ LLC, Novosibirsk, Russia) in a glass protecting tube and a Pt100-type resistance temperature detector (TS-1288 F/11; ‘Elemer’ LLC, Podolsk, Russia) connected to a multichannel thermometer (TM 5103; ‘Elemer’ LLC, Podolsk, Russia). The atmospheric pressure was detected by a barometer (BTKSN-18; Technical Specification No. 1-099-20-85, ‘UTYOS’ JSC, Ulyanovsk, Russia). The general scheme of the test facility is shown in Figure 6.

The hydrogen volume data sets at standard conditions (Standard DIN 1343: 101,325 Pa, 0 °C) were calculated via the ideal gas law using the recoded mass, temperature, and atmosphere pressure data. Hydrogen yield (in %) represented the calculated hydrogen volume values divided by the hydrogen volume corresponding to the entire oxidation of aluminum in the sample. Each experiment was repeated three times, and the corresponding three data sets were used for the calculation of the mean values (kinetic curves) and standard deviations (error bars).

## 4. Conclusions

In the present research, different parameters for the manufacture of composite hydro reactive materials and their hydrogen generation performance were tested. The key concept for the material elaboration consisted in the mechanical coupling of Ag with Al via high-energy ball milling since Ag was expected to promote vigorous galvanic corrosion of Al in aqueous saline media (from an aggressive AlCl_3_ solution to a ‘moderate’ CaCl_2_ and a ‘mild’ NaCl brines) with intensive hydrogen evolution. For better clarification of the specific Ag effect and elimination of potential contributions from impurities out of consideration, chemically pure (99.99%) aluminum swarf was used as the basic material.

The summarization of the study results delivered the following key findings. Swarf chips of high-purity aluminum were too ductile to be effectively ground into powder via ball milling with heavy tungsten carbide balls. Overcoming that obstacle involved the implementation of additives for accelerating particle size reduction and preventing excessive agglomeration of aluminum pieces. The viable combinations for obtaining fine powders were the introduction of toluene as a process control agent together with LiCl or Ga serving as ‘grinding agents’. Experiments with the Al–LiCl, Al–Ga, Al–LiCl–Ag, and Al–Ga–Ag in the AlCl_3_ solution at 60 °C provided notable outputs: for all samples (excepting Al–LiCl), nearly 60–80% hydrogen yields were achieved in less than 2 min., since the reaction progress was quite fast. The final hydrogen yields, however, fell below 90%. Such an ‘underperformance’ was attributed to the partial oxidation of aluminum and its contamination with WC during ball milling, which resulted in decreased contents of ‘disposable’ aluminum in the powders. The results of the XRD analysis supported the fact that the samples containing Ga had higher contents of aluminum oxide as compared to those with LiCl. Such an effect was explained by the shielding of the aluminum particles’ surfaces with the incrusted salt pieces, which was proved by the XPS data (high contents of Li and Cl on the surface of the Al–LiCl–Ag sample). The supreme reaction rates for the samples containing Ag resulted from the formation of ‘microgalvanic cells’ between aluminum and far ‘nobler’ silver, which caused intensive galvanic corrosion of Al with hydrogen evolution. The lower reaction rates for the samples containing LiCl were ascribed to the mentioned particles’ surface shielding by salt particles (whose dissolution took some time) and, presumably, to a larger content of oxides on the sample’s surface, according to the XPS data. Trials with the most reactive Ag-modified powder sorts in both CaCl_2_ and NaCl brines under the same temperature did not deliver mentionable results. At an elevated temperature of 80 °C, the NaCl brine still underperformed, while in the case of CaCl_2_, the reaction proceeded rather fast in the beginning. That trend, however, became decelerating before achieving high outputs, and after 3 h of experiment, less than 50% of hydrogen was released for both samples. In the case of the AlCl_3_ solution, fast reaction progress was attributed to its high hydrolysis performance, which delivered a good deal of chloride ions, which provided the formation of complex compounds with a high solubility that ensured their effective removal from the samples’ surfaces. In the case of the CaCl_2_ ‘brine’ hydrolysis, a far smaller amount of chlorine ions was formed, and their possible effect consisted in clustering near the Al lattice imperfections and local destruction of the passivation layer by pitting corrosion mechanisms.

Since, in addition to its ductility unfavorable to particle size reduction via ball milling, chemically pure aluminum was known for its strong corrosion resistance, setting up its reaction with vigorous hydrogen evolution was a challenging task in the first place. Although the tested Ag-modified samples provided hydrogen generation yields lower than expected, they demonstrated quite impressive reaction rates. Therefore, such materials have the potential for improvement.

## Figures and Tables

**Table 1:** Elemental compositions for the selected points of the original and resulting samples.

Sample	Spectra No.	Al	O	Cl	Ga	Ag	W
Al swarf	42	99.5 ± 0.2	0.5 ± 0.2	-	-	-	-
	44	99.2 ± 0.2	0.8 ± 0.2	-	-	-	-
Al–Li	37	87.3 ± 0.3	3.9 ± 0.3	8.7 ± 0.1	-	-	-
	41	64.2 ± 0.4	9.3 ± 0.4	23.8 ± 0.2	-	-	2.7 ± 0.4
Al–Li–Ag	23	68.2 ± 0.4	10.9 ± 0.4	12.3 ± 0.2	-	8.6 ± 0.2	-
	25	46.6 ± 0.3	12.1 ± 0.4	12.4 ± 0.1	-	28.9 ± 0.3	-
Al–Ga	13	87.6 ± 0.5	8.4 ± 0.3	-	0.8 ± 0.2	-	3.2 ± 0.4
	15	88.6 ± 0.4	10.4 ± 0.3	-	1.0 ± 0.2	-	-
Al–Ga–Ag	7	79.8 ± 0.5	6.8 ± 0.3	-	0.6 ± 0.2	11.6 ± 0.3	1.2 ± 0.4
	8	77.6 ± 0.4	10.7 ± 0.4	-	0.6 ± 0.2	11.1 ± 0.3	-

**Table 2:** Hydrogen yields and maximum evolution rates for a ‘strong solution’.

Sample	Solution	Temperature, °C	Hydrogen Yield, %	Maximum Hydrogen Evolution Rate, mL/g/min.
Al–Ga–Ag	AlCl_3_	60	80.2 ± 0.5	792
Al–Ga			76.7 ± 0.7	588
Al–LiCl–Ag			84.6 ± 0.2	586
Al–LiCl			86.8 ± 1.4	183

**Table 3:** Hydrogen yields and maximum evolution rates for a ‘moderate brine’.

Sample	Solution	Temperature, °C	Hydrogen Yield, %	Maximum Hydrogen Evolution Rate, mL/g/min.
Al–Ga–Ag	CaCl_2_	80	31.8 ± 1.9	72
Al–LiCl–Ag			46.7 ± 2.1	89

## Data Availability

Data are contained within the article.

## Appendix A

The EDX analysis results for the aluminum alloy swarf and ball-milled composite powders are shown in Figure A1. In the SEM images, all inspected points were marked, while the attached EDX spectra were limited by the selected, most representative spots because many of them had quite identical elemental compositions.
